# First report of successful pregnancies after treatment with alpelisib for *PIK3CA*-related overgrowth spectrum

**DOI:** 10.1038/s41431-025-01885-y

**Published:** 2025-06-06

**Authors:** Gabriel Morin, Antoine Fraissenon, Caroline Chopinet, Estelle Balducci, Sophie Kaltenbach, Patrick Villarese, Vahid Asnafi, Laurent Guibaud, Guillaume Canaud

**Affiliations:** 1 Université Paris Cité, Paris, France; 2 INSERM U1151, Institut Necker-Enfants Malades, Paris, France; 3 Unité de Médecine Translationnelle et Thérapies Ciblées, Hôpital Necker-Enfants Malades, AP-HP, Paris, France; 4 Service d’Imagerie Pédiatrique, Hôpital Femme-Mère-Enfant, HCL, Bron, France; 5 CREATIS UMR 5220, Villeurbanne, France; 6 Service de Radiologie Mère-Enfant, Hôpital Nord, Saint Etienne, France; 7 Service de Physiologie & Explorations Fonctionnelles Cardiovasculaires, CHU de Lille, Lille, France; 8 Laboratoire d’Oncohématologie, Hôpital Necker-Enfants Malades, AP-HP, Paris, France

**Keywords:** Medical research, Diseases

## Abstract

Alpelisib is a selective PI3Kα inhibitor approved for treating *PIK3CA*-related overgrowth spectrum (PROS), a group of rare malformation disorders. Given that PI3Kα is a ubiquitous protein involved in cell proliferation, understanding the long-term impact of alpelisib on fertility is of critical importance. Here, we report the favorable outcomes of three pregnancies in PROS patients after prolonged treatment with alpelisib. Although disease progression was observed in all three patients during pregnancy, vascular malformations remained sensitive to alpelisib without evidence of secondary resistance upon resuming treatment. In conclusion, we provide the first evidence that alpelisib does not appear to affect fertility in female patients with PROS.

## Introduction

Phosphatidylinositol 3-kinase α (PI3Kα) is a ubiquitous protein kinase that controls cell growth, proliferation, and survival [1]. Somatic variants of *PIK3CA*, the gene encoding for the catalytic subunit of PI3Kα, are found in breast cancer and in *PIK3CA*-related overgrowth spectrum (PROS), a group of congenital malformation syndromes that involve vascular, adipose, cutaneous and skeletal malformations, along with segmental overgrowth [2]. Recently, alpelisib, a specific PI3Kα inhibitor, was approved for treating *PIK3CA*-mutant metastatic breast cancer and PROS. Alpelisib has been associated with several adverse events, including diarrhea and nausea, alopecia, abnormal glucose metabolism (from hyperglycemia to diabetes) and rash [3, 4]. However, little is known regarding the long-term effects of alpelisib, especially on fertility. Animal studies showed that alpelisib in female rats was associated with variations in estrus cycle, vaginal/uterine atrophy, increased embryonic loss and embryonic skeletal malformations (https://ec.europa.eu/health/documents/community-register/2020/20200727148490/anx_148490_en.pdf). Given the embryonic toxicity of alpelisib, patients under treatment are advised to use effective contraception. However, despite the large use of alpelisib worldwide, no data are currently available regarding its impact on fertility. In addition, the consequences of discontinuing alpelisib during pregnancy on disease progression and treatment sensitivity upon reinitiation is unknown. Here, we report the successful outcomes of three pregnancies in three primiparous PROS patients who underwent prolonged treatment with alpelisib, showing no evidence of acquired resistance upon resumption of treatment.

## Methods

### Patients

This study was conducted on 3 patients who were followed at *Hôpital Necker Enfants Malades, CHU de Lille* and *Hôpital Mère Enfants de Lyon*, following procedures previously described elsewhere [5]. Treatment was administered according to the protocol approved by the French Regulatory Agency (reference numbers: 1013795, 1098458 and 1098458) and the Novartis Managed Access Program. Written informed consent was obtained from all patients. Alpelisib was compassionately offered by Novartis. Patients received 250 mg/day. Alpelisib was taken orally every morning during breakfast. Patients were assessed at regular intervals as previously reported [5–8]. Adverse events were graded according to the Common Terminology Criteria for Adverse Events [CTCAE], version 4.03, and coded by preferred term using the Medical Dictionary for Regulatory Activities [MedDRA], version 24.0.

### Imaging

Patients underwent full body magnetic resonance imaging (MRI) prior to alpelisib introduction, before alpelisib withdrawal, then before alpelisib reintroduction. MRI examination was performed using T1, T2 and T2 with fat suppression weighted imaging sequences. Volumetric evaluation on MR exams was performed with 3D Slicer software with manual segmentation tools [9]. Volumes were calculated by summing images based on 2D contours and slice thickness.

## CASES

**Fig. 1:**
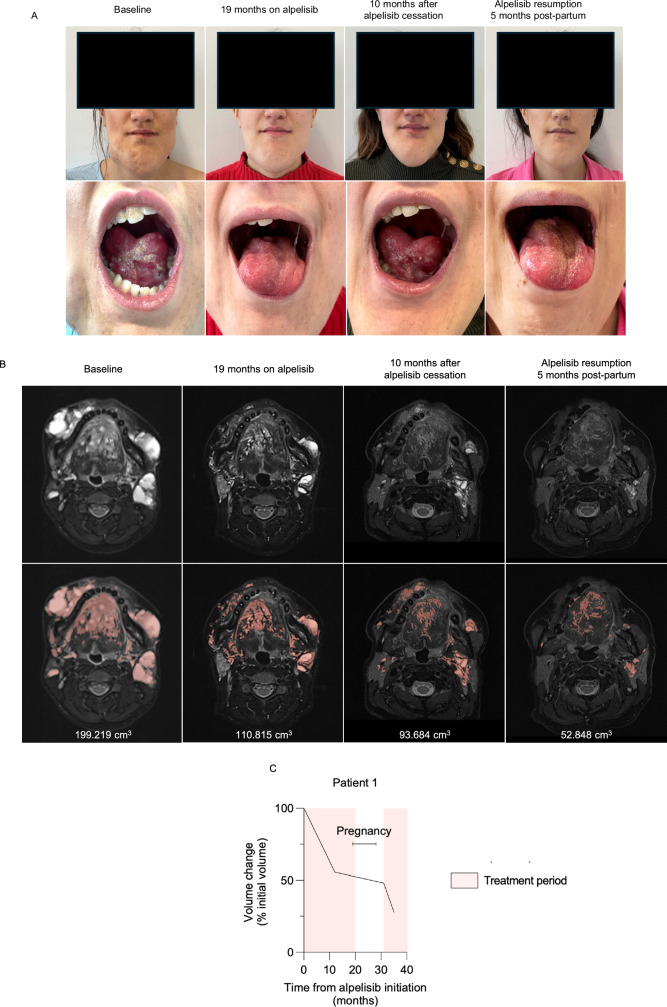
Patient 1 following alpelisib withdrawal and reintroduction. **A** Representative pictures of patient 1 over time. **B** Representative transversal MRI T2 fat sat sequences of patient 1 over time. **C** Magnetic resonance volumetric quantification of the malformation over time.

Patient 1 was 21 years old when she began treatment with alpelisib (250 mg/d) for severe and complex *PIK3CA*-related facial venous-lymphatic malformations (Fig. 1A). She experienced multiple inflammatory flares each year, requiring treatment with antibiotics and steroids, as well as recurrent buccal bleeding and severe pain when eating. Starting at 4 days old, she underwent over 20 interventional procedures, including surgeries and sclerotherapies, with subsequent worsening of the vascular malformations. After initiating alpelisib, she showed good clinical response including pain reduction, cessation of inflammatory flares and bleeding, and a decrease in the vascular malformation volume as assessed by MRI (Fig. 1A, B). After 19 months on alpelisib, despite being advised to maintain effective contraception, she became pregnant while still on the treatment. She discontinued alpelisib approximately 26 days after conception. Due to increased nuchal translucency, she underwent a trophoblast biopsy which confirmed a normal karyotype. Subsequent ultrasound follow-ups revealed neither structural nor biometric fetal anomalies. She delivered by vaginal route at 40 weeks of amenorrhea without complications (Supplementary Table 1). The neonate exhibited normal growth and is now 20 months old, with no evidence of alpelisib-related toxicity from early in utero exposure. During this time, the vascular malformations progressed clinically with recurrent inflammatory flares (Fig. 1A). Since the patient did not breastfeed, alpelisib (250 mg/day) was resumed two months postpartum, and an MRI was performed prior to reinitiating treatment, approximately 10 months after its withdrawal. Clinical and MRI reevaluation showed a reduction in the vascular malformation volume with no indications of acquired resistance to treatment (Fig. 1A–C).

**Fig. 2:**
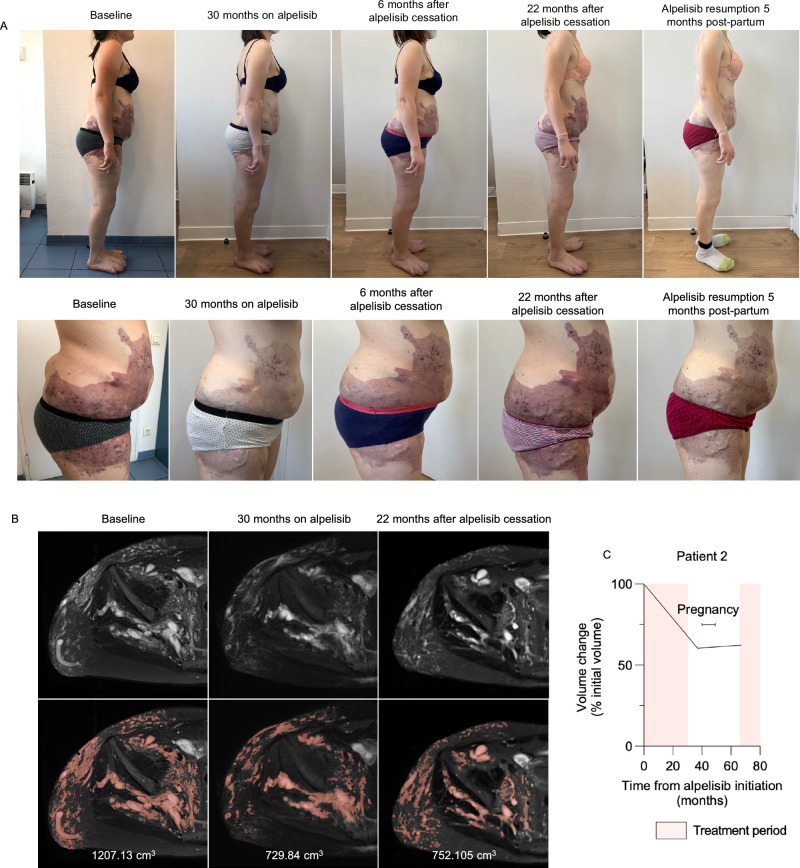
Patient 2 following alpelisib withdrawal and reintroduction. **A** Representative pictures of patient 2 over time. **B** Representative transversal MRI T2 fat sat sequences of patient 2 over time. **C** Magnetic resonance volumetric of the malformation over time.

Patient 2 had CLOVES syndrome with an extensive *PIK3CA*-related capillary-venous-lymphatic malformation of the right trunk, flank, pelvis and thigh, as well as a cystic lumbar lymphangioma (Fig. 2A). She exhibited limb length discrepancy due to overgrowth of the right lower limb and experienced multiple recurrent thrombotic events caused by localized intravascular coagulation. Additionally, she had familial hexadactyly of both hands, which was unrelated to the *PIK3CA* variant. She underwent more than 30 surgical procedures, including vascular malformation resection and orthopedic interventions. Progression of the vascular malformation led to sciatic pain, requiring treatment with pregabalin and the use of a wheelchair. She received sirolimus for one year with transient efficacy on pain but had recurrent deep venous thrombosis despite prophylactic treatment with enoxaparin. At the age of 25, she started alpelisib (250 mg/d), resulting in both clinical and radiological efficacy (Fig. 2A, B). After 30 months of treatment, alpelisib was discontinued to allow for conception. She became pregnant spontaneously ten months after treatment cessation, at the age of 28. Within a few weeks of alpelisib discontinuation, she showed early signs of disease progression, including intense pain, difficulty walking (necessitating wheelchair use), inflammatory flares with oozing, and multiple deformations. Nevertheless, she decided to continue with the pregnancy.

During pregnancy, she received folic acid, enoxaparin and pregabalin, as well as anti-D immunoglobulins due to maternal-fetal rhesus incompatibility. At seven weeks of amenorrhea, she experienced paucisymptomatic COVID19, which resolved without complications. Prenatal sonographic exams showed normal fetal biometry and absence of any structural anomalies except for a post-axial familial hexadactyly of both hands. She delivered vaginally at 37 weeks of amenorrhea without complications (Supplementary Table 1). Apart from bilateral hexadactyly, the neonate exhibited no abnormalities, with normal growth and development observed during the first 24 months of life, as of the latest follow up. However, the vascular malformation progressed following treatment interruption until breastfeeding cessation (Fig. 2A–C). She then resumed alpelisib (250 mg/d). Following the implantation of a contraceptive intrauterine device, she reported skin and vaginal dryness (grade 2) which resolved after reducing the alpelisib dose to 125 mg/d. A recurrent inflammatory flare a few weeks later prompted an increase in the dose to 175 mg/d. Three months after resuming alpelisib, the vascular malformation discolored and clinically decreased in size (Fig. 2A, B).

Patient 3 had *PIK3CA*-related overgrowth syndrome associated with an extensive retroperitoneal lymphatic malformation, multiple lipomatosis, left hemihypertrophy, scoliosis, and obesity. She underwent 16 surgical procedures starting at the age of 11. At 22, she began alpelisib (250 mg/d) due to progression of the voluminous vascular malformation which compressed neighboring organs and caused severe pain, fatigue, and limited walking capacity. Alpelisib was associated with symptoms improvement and reduction in the vascular malformation volume as assessed by MRI (Fig. 2A, B). After 23 months, she spontaneously discontinued both alpelisib and contraception and became pregnant three months later. During pregnancy, fetal biometric measures remained within normal ranges without any structural anomaly as assessed by ultrasound (Supplementary Table 1). At 40 weeks of amenorrhea, she delivered vaginally following prolonged spontaneous rupture of membranes, which required labor induction and amoxicillin therapy. Fetal extraction was difficult, necessitating the use of ventouse suction cups, spatulas, then forceps. The neonate had an Apgar score of 1/10 at birth, with bradycardia and acidosis in the cord blood, but improved to 10/10. Electroencephalographic assessment revealed global hypovoltage with a few temporal slow spikes during sleep with normal reactivity, evocative of mild neurological injury. The neonate experienced transient peripheral facial paralysis and dermabrasions due to extraction methods. He developed jaundice without other neurological complications, and intensive phototherapy was successfully performed. Subsequent neurological development was normal, and growth charts were within the normal range. The baby is now 14 months old.

**Fig. 3:**
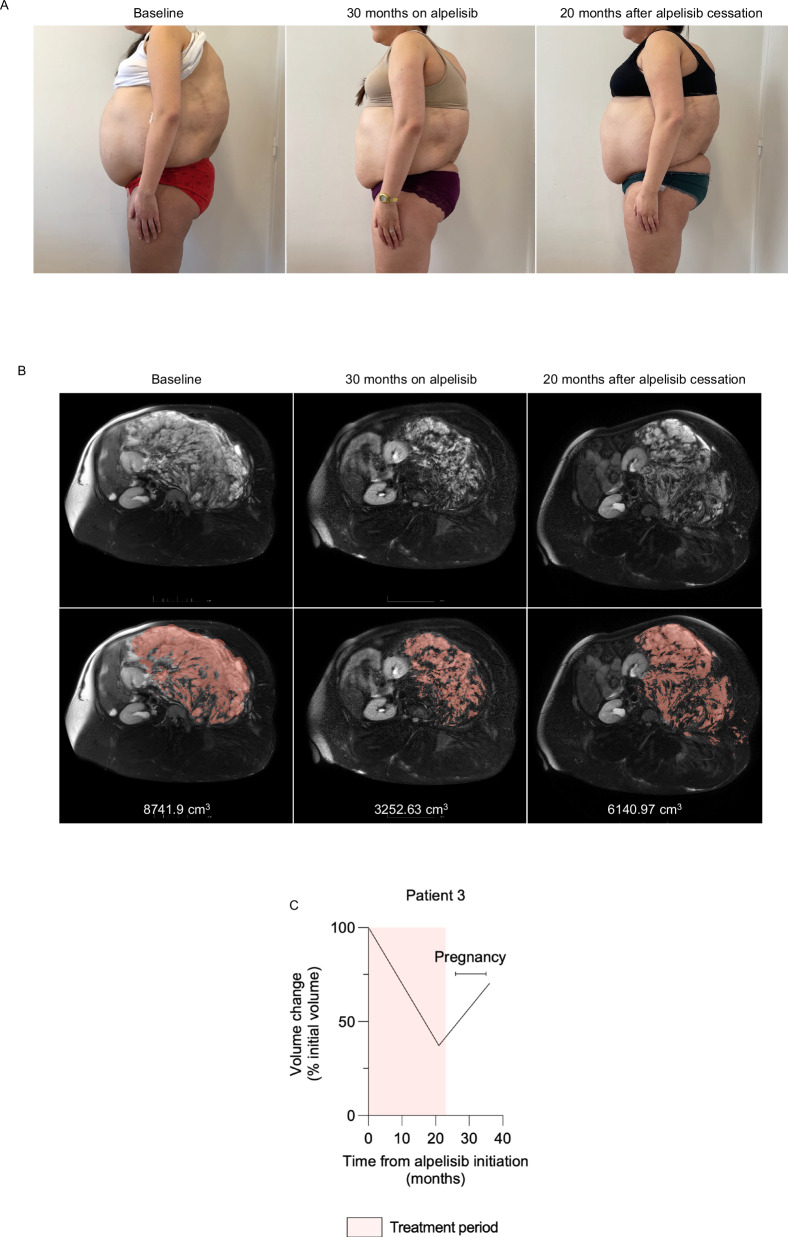
Patient 3 following alpelisib withdrawal and reintroduction. **A** Representative pictures of patient 3 over time. **B** Representative transversal MRI T2 fat sat sequences of patient 3 over time. **C** Magnetic resonance volumetric of the malformation over time.

The patient demonstrated symptoms of PROS progression, with regrowth in all affected areas (Fig. 3A–C). She was scheduled to resume alpelisib a few months after delivery due to malformations-related pain; however, she was found to be two weeks pregnant again, contraindicating reintroduction of the drug. This second pregnancy is ongoing without any notable issues.

## Discussion

Alpelisib is approved and used in clinical practice for the treatment of both PROS and hormone receptor-positive, HER2-negative, PIK3CA-mutated metastatic breast cancer (in combination with endocrine therapy) [3]. However, long-term impact of alpelisib on fertility is not known, which is of particular interest in young patients with PROS who may be treated for years. We report three cases of PROS patients who developed spontaneous pregnancies after prolonged treatment with alpelisib and delivered healthy neonates, without evidence of infertility. Notably, one of these pregnancies was unplanned, with accidental embryonic exposure to alpelisib. The medication was discontinued as soon as pregnancy was suspected. Fortunately, no drug-related embryotoxicity was observed. Nevertheless, patients of reproductive age should be reminded at every consultation of the requirement to use effective contraception while on alpelisib.

These three cases lead us to make several preliminary observations that require further investigation before drawing formal conclusions. First, prolonged exposure to high doses of alpelisib did not appear to impair fertility in these 3 patients. Second, no drug-related embryotoxicity was observed. Third, symptoms related to *PIK3CA* variants rapidly recurred following alpelisib interruption, highlighting that prolonged treatment does not cure the underlying genetic defect. Clinical worsening might be explained by the recurrence of cell growth and proliferation in the affected areas upon treatment withdrawal. Finally, it is reassuring to note that alpelisib reintroduction effectively alleviated symptoms and reduced the size of the malformations, supporting the absence of acquired resistance to the treatment.

Overall, a “stop-and-go” approach of alpelisib may provide a window for conception without compromising disease sensitivity to treatment. Patients should be advised that disease symptoms and malformation growth may recur, sometimes rapidly, following treatment discontinuation. It is therefore essential to closely monitor pregnancies in PROS patients.

## Supplementary information

Supplementary Table

## Data Availability

Data sharing not applicable to this article as no datasets were generated or analyzed during the current study.
