# Supplementary material for: First report of successful pregnancies after treatment with alpelisib for PIK3CA-related overgrowth spectrum
Source: Eur J Hum Genet. 2025 Jun 6;33(8):1066–71. doi: 10.1038/s41431-025-01885-y (PMC12322174; doi:10.1038/s41431-025-01885-y)
Supplement: Supplementary file 1 — Supplementary Table [file 41431_2025_1885_MOESM1_ESM.docx]

|  | PIK3CA variant  Genomic mutation ID  CDS mutation  AA mutation  ACMG classification  Variant allele frequency | Previous pregnancies | Age at introduction of alpelisib | Embryonic exposure duration (days) | Age at pregnancy | Maternal complications | Obstetrical complications | Delivery route | Neonatal complications | Delivery term  (weeks + days of amenorrhea) | Birth weight/height (cm) | APGAR score  (0/1 min) |
| --- | --- | --- | --- | --- | --- | --- | --- | --- | --- | --- | --- | --- |
| Patient 1 | Postzygotic  COSV55873227  c.1624G>A  p.Glu542Lys  Class 5  8% | None | 21 | 26 | 22 | Infected tooth avulsion  Abdominal pain at 35 weeks of amenorrhea without obstetrical complication | Increased nuchal translucency  Trophoblast biopsy: normal karyotype | Vaginal | None | 40+4 | 3940/51 | 10/10 |
| Patient 2 | Postzygotic  COSV55873239  c.1633G>A  p.Glu545Lys  Class 5  6% | None | 25 | 0 | 28 | Simple COVID19 | Rhesus incompatibility | Vaginal | None | 37 | 3100/50 | 10/10 |
| Patient 3 | Postzygotic  COSV55873195 c.3140A>G  p.His1047Arg  Class 5  3% | None | 22 | 0 | 24 | Delivery hemorrhage from atonic uterus  Resolution after uterine revision, uterine massage, IV oxytocin, tranexamic acid and cefazolin | Prolonged spontaneous rupture of membranes  Stimulation of delivery  Mechanical extraction | Vaginal | Bradycardia  Spontaneously resolutive acidosis  Jaundice (intensive phototherapy) | 40+1 | 3470/50 | 1/10 |

**Supplementary Table 1: Patients characteristics**
